# ASSOCIATIONS BETWEEN OBJECTIVE HEARING FUNCTION AND SUBJECTIVE VIEWS OF AGING

**DOI:** 10.1093/geroni/igae098.3267

**Published:** 2024-12-31

**Authors:** Jana Koch, Brooke Brady, Kaarin Anstey

**Affiliations:** University of New South Wales, Sydney, New South Wales, Australia; University of New South Wales, Sydney, New South Wales, Australia; University of New South Wales, Sydney, New South Wales, Australia

## Abstract

Subjective views of aging have been associated with positive health-promoting behaviours and well-being. While age-related changes are considered fundamental to views of aging, the role of declining hearing function in shaping these age views remains a largely unexplored area. Within the scope of this study, we hypothesized that poor hearing function would contribute to less favorable views of aging, as measured on multiple related constructs. Data were obtained from the app-based, longitudinal ‘Labs without Walls’ study, including 184 participants with a mean age of 55.6 years (17.1 SD). Subjective views of aging were measured by the Self-perceptions of aging scale, Expectations regarding aging scale (with subscales on physical health, mental health, and cognitive function), subjective age, and ideal age. Hearing function was measured on a validated app-based hearing task, and a pure-tone average was calculated in the better-hearing ear on frequencies 500, 1000, 2000, and 4000 Hz. Structural equation modelling was employed to examine the unique contributions of hearing function on subjective views of aging while controlling for chronological age, sex-at-birth, loneliness, cognition and socioeconomic status. As hypothesized, poor hearing function was associated with negative expectations regarding aging, especially regarding maintaining cognitive function and physical health. Hearing function was also associated with an older ideal age. However, poor hearing function was not predictive of any other constructs of subjective views of aging. These findings highlight that hearing loss affects expectations of the aging process. Future interventions should examine whether addressing these negative expectations could lead to improved hearing health behaviors.

